# Oxidative Stress in Zebrafish (*Danio rerio*) Sperm

**DOI:** 10.1371/journal.pone.0039397

**Published:** 2012-06-19

**Authors:** Mary Hagedorn, Megan McCarthy, Virginia L. Carter, Stuart A. Meyers

**Affiliations:** 1 Department of Reproductive Sciences, Smithsonian Conservation Biology Institute, Washington, District of Columbia, United States of America; 2 Hawaii Institute of Marine Biology, University of Hawaii, Kaneohe, Hawaii, United States of America; 3 Department of Anatomy School of Veterinary Medicine, University of California at Davis, Davis, California, United States of America; New England Biolabs, Inc., United States of America

## Abstract

Laboratories around the world have produced tens of thousands of mutant and transgenic zebrafish lines. As with mice, maintaining all of these valuable zebrafish genotypes is expensive, risky, and beyond the capacity of even the largest stock centers. Because reducing oxidative stress has become an important aspect of reducing the variability in mouse sperm cryopreservation, we examined whether antioxidants might improve cryopreservation of zebrafish sperm. Four experiments were conducted in this study. First, we used the xanthine-xanthine oxidase (X-XO) system to generate reactive oxygen species (ROS). The X-XO system was capable of producing a stress reaction in zebrafish sperm reducing its sperm motility in a concentration dependent manner (P<0.05). Second, we examined X-XO and the impact of antioxidants on sperm viability, ROS and motility. Catalase (CAT) mitigated stress and maintained viability and sperm motility (P>0.05), whereas superoxide dismutase (SOD) and vitamin E did not (P<0.05). Third, we evaluated ROS in zebrafish spermatozoa during cryopreservation and its effect on viability and motility. Methanol (8%) reduced viability and sperm motility (P<0.05), but the addition of CAT mitigated these effects (P>0.05), producing a mean 2.0 to 2.9-fold increase in post-thaw motility. Fourth, we examined the effect of additional cryoprotectants and CAT on fresh sperm motility. Cryoprotectants, 8% methanol and 10% dimethylacetamide (DMA), reduced the motility over the control value (P<0.5), whereas 10% dimethylformamide (DMF) with or without CAT did not (P>0.05). Zebrafish sperm protocols should be modified to improve the reliability of the cryopreservation process, perhaps using a different cryoprotectant. Regardless, the simple addition of CAT to present-day procedures will significantly improve this process, assuring increased and less variable fertilization success and allowing resource managers to dependably plan how many straws are needed to safely cryopreserve a genetic line.

## Introduction

In the past decade, laboratories around the world have produced tens of thousands of mutant and transgenic zebrafish lines. As with mice, maintaining all of these valuable zebrafish genotypes is expensive, risky, and beyond the capacity of even the largest stock centers [1]. Our long-term goal is to preserve genetic resources from aquatic model organisms, specifically zebrafish, that are vital to advancing biomedical research and knowledge. Although conventional cryopreservation is a proven method for long-term, safe storage of genetic material, protocols used by zebrafish researchers are not standardized and yield inconsistent results with high post-thaw variability [2]–[6], thereby putting the security of many genotypes in individual laboratories and stock centers at great risk.

Fish have similar homeostatic enzyme systems to those reported in many mammalian systems [7]. Specifically, reactive oxygen species (ROS) are produced during respiration and can interact with organic substances. They can also be produced by intracellular enzyme systems like NADPH oxidase and cytoplasmic xanthine [7]. When these reactive oxygen species overcome the buffering capacity of the cell, the cell then enters oxidative stress, potentially leading to damage of DNA/RNA, proteins and lipids. Several cell adaptations exist which counteract the negative effects of oxidative stress, such as a thiol reducing buffer composed of glutathione and thioredoxin and enzymes to remove reduced oxygen species, such as catalase (CAT), superoxide dismutase (SOD) and glutathione peroxidase. However, zebrafish somatic cells have electrophile responsive genes that encode proteins that inactivate oxidants, thus avoiding oxidative stress [8], [9] When zebrafish are exposed to oxidative stress, this induces a genetic pathway that produces antioxidants to help ward off the negative aspects of the stress [8], [9]. However, zebrafish sperm cells are terminal haploid cells with tightly coiled DNA. These cells do not have access to their natural anti-oxidant pathway simply because this terminal cell type does not transcribe DNA or translate RNA.

An increase in ROS has been linked to abnormal or damaged spermatozoa [10]–[14], and this may be especially true in fish. For example, duroquinone induced ROS in carp spermatozoa that caused DNA damage in the sperm and subsequently impaired reproductive success [15]. The xanthine-xanthine oxidase (X-XO) system is one of several ROS generating systems frequently used in biology to investigate the cytotoxic effects of superoxide anion and hydrogen peroxide. Generation of ROS *in vitro* by the (X-XO) system results in a reduction in sperm motility [16]–[19], viability [16], [20], ionophore-induced acrosome reaction [18], [21] and sperm-oocyte fusion [18], [22]. Hydrogen peroxide, in contrast to superoxide anion, is more stable, less polar, and can readily cross the plasma membrane [23]. Consequently, hydrogen peroxide appears to be the primary ROS responsible for oxidative damage to spermatozoa *in vitro* [16]–[19], [22], [24], [25], and membrane lipid peroxidation is believed to be an important mechanism of action [10], [18], [26]. Spermatozoa are particularly susceptible to lipid peroxidation because they contain high concentrations of unsaturated fatty acids [27] and, as terminally differentiated cells, have limited repair mechanisms [28], [29]. As a consequence of lipid peroxidation, the plasma membrane loses the fluidity and integrity it requires for participation in the membrane fusion events associated with fertilization [26], [30], [31].

Spermatozoa and seminal plasma possess a number of enzymes and low molecular weight antioxidants that scavenge ROS in order to prevent cellular damage. Spermatozoa contain a limited volume of cytoplasm and are predominantly dependent upon the antioxidant support of seminal plasma [32]–[34]. Sperm preparation for cryopreservation involves the removal of seminal plasma, thereby increasing the susceptibility of spermatozoa to oxidative stress. In addition, research suggests that the antioxidant activity of the spermatozoa themselves may be decreased by cryopreservation [35]–[37]. Furthermore, freeze-thawing of equine spermatozoa [14] and cryopreservation of human [38] and bovine [39], [40] spermatozoa was associated with an increase in reduced oxygen species generation. Oxidative damage to DNA in cryopreserved bovine sperm had a high negative correlation with pregnancy rates [41]. The addition of enzyme scavengers or antioxidants to sperm preparations *in vitro* has been successful at counteracting the effects of oxidative stress on sperm motility, viability, lipid peroxidation, sperm-oocyte fusion and DNA fragmentation [18], [22], [42]–[46].

Because reducing oxidative stress has proven to be an important aspect of reducing the variability in mouse sperm cryopreservation [47]–[49], we examined whether this might show the same types of improvements for zebrafish sperm. It was not known whether antioxidants that scavenge ROS in order to prevent cellular damage would be beneficial for zebrafish sperm cryopreservation. In order to examine this issue, four experiments were conducted in this study. In Experiment 1, the X-XO system was established with zebrafish sperm and levels of ROS production were determined. In Experiment 2, three antioxidant substances, SOD, CAT and vitamin E were examined to determine whether they could mitigate the exogenous stress produced by the X-XO system. These antioxidants are commonly found in fish tissue [50]. In Experiment 3, we cryopreserved zebrafish sperm [6], evaluated the level of ROS production, and used CAT in pre-freeze and post-thaw samples to determine whether these antioxidants would reduce stress and improve motility and viability. Finally, in Experiment 4, we analyzed whether antioxidants might improve pre-freeze motility of three commonly used cryoprotectants [2]–[6]. We hypothesized that a potent antioxidant could be used to improve post-thaw motility and reduce variability in this valuable biomedical model. In future applications of cryopreservation, zebrafish sperm protocols could be modified to improve the reliability of the cryopreservation process and allow resource managers to reliably plan how many straws are needed to safely cryopreserve a line.

## Materials and Methods

### Maintenance of Animals

Fish were housed in standard microcosms (Aquatic Ecosystems, Apopka, FL) that have independent, water, temperature and waste management with sensors on each rack constantly monitoring the pH, temperature and conductivity of the water. The Zebrafish International Resource Center (ZIRC) at the University of Oregon has prepared detailed user manuals that describe standard operating procedures [51]. We followed their recommended facilities operations including care and maintenance of adults, breeding and obtaining gametes and embryos, record keeping, sending and receiving fish from other laboratories, quarantine and other procedures relating to disease control, and euthanasia. Briefly, AB wild-type fish were obtained from stocks at ZIRC at approximately 4 months of age. They were maintained in recirculating dechlorinated systems at 26–28°C with an artificial light cycle (14 h light; 10 h dark). Feeding schedule consisted of twice-daily provision of live brine shrimp (*Artemia* nauplii) and “Master Mix” dry food (a combination of Nelson’s Silver Cup Tropical No. 1, Spirulina Flake, Golden Pearl and Cyclopeeze, see Westerfield [51] for details). Fish were examined daily for any signs of stress or disease and any fish in poor health were removed from the experimental group. Condition factors were standardized to use males of 5 to 10 months old with a condition factor of 1.5 to 2.5. Condition factor is defined as K = 100,000×weight/(standard length)^3^ [52]. All care and welfare for the animals met NIH animal care standards. Full details of the study approval are listed with the Smithsonian CRC-IACUC (approval ID #06-19) and the University of Hawaii, Hawaii Institute of Marine Biology IACUC (protocol ID# 06-022).

### Collection of Germplasm

The procedure used to obtain sperm was gentle abdominal squeezing of mature males [51]. The afternoon prior to squeezing, males were chosen and removed from mixed-sex tanks and placed into a single tank as a group. Briefly, males were immersed in a solution of tricaine methane sulfonate (MS-222) made according to Westerfield [51] until gill movements have slowed (∼30 sec). To collect sperm, males were rinsed with clean aquarium water, excess water was removed by placing fish on a KimWipe, so that standard length and weight could be taken and recorded. Fish were then placed on a slit, damp sponge and oriented with their dorsal surface down. While viewed under a dissecting microscope, the anal fin area was dried and gentle pressure was exerted using forceps to squeeze both sides of the fish at a point just anterior to the pelvic fins. The sperm was collected with a 1-ml capillary tube, amount recorded and then placed into an Eppendorf tube on ice to await more sperm, if pooling, or to be expressed into chilled buffer. Fish were returned to a recovery tank for observation after squeezing. Sperm was then diluted to appropriate concentrations for each experiment into Hanks balanced salt solution (HBSS) at 300 mOsm/kg (0.137 M NaCl, 5.4 mM KCl. 1.3 mM CaCl. 1.0 mM MgSO_4_, 0.25 mM Na_2_HPO_4_, 4.2 mM NaHCO_3_ and 5.55 mM glucose, pH 7.2) to maintain the sperm and prevent activation. Sperm was held on ice until used in experiments. Generally, ejaculate volume of a single male was ∼1 µl at 10^10^ cells/ml [53]. Depending on the number of treatments, usually a sample consisted of pooled sperm from 3 to 5 males, diluted ∼1∶1000 with HBSS, then tested across the array of treatments. If more treatments were performed, the number of males in the pooled sample was increased. Throughout the study, the sperm concentration present in all treatment vials was 5×10^6^ to 1×10^7^ cells/ml.

### Chemicals

Superoxide Dismutase (SOD), Xanthine Oxidase (XO), α-Tocopherol (Vitamin E dissolved in 95% methanol), dimethylacetamide (DMA), dimethylformamide (DMF) and catalase (*Aspirgillus niger*, Sigma C3515, CAT), were obtained from Sigma Chemical Co. (St. Louis, MO, USA). Xanthine was purchased from Calbiochem (La Jolla, CA, USA). Dihydroethidium (DHE), 4,4-difluro-5-(4-phenyl-1,3-butadienyl)-4-bora-3a,4a-diaza-s-indacene-3-undecanoic acid (C_11_-BODIPY^® 581/591^), propidium iodide and SYTOX® Green, were obtained from Invitrogen (Eugene, OR, USA). All percent solutions were made up vol/vol in HBSS, unless otherwise stated.

### Cellular Analysis

We measured the number of membrane intact cells and superoxide production within samples with two types of fluorescent stain pairs (DHE and SYTOX® Green) and (BODIPY® and propidium iodide) measured on a flow cytometer (see below).

DHE indicates the presence of general superoxide production because it permeates the cell and exhibits a blue-fluorescence in the cytosol. When oxidized, however, it intercalates within the cell’s DNA, staining its nucleus a bright fluorescent red. SYTOX® Green nucleic acid stain only penetrates cells with compromised membranes and does not cross membranes of living cells. After various treatments (see details below), DHE was added to each sample (2 µM for 10 min) followed by SYTOX® Green (0.05 µm for 5 min).

BODIPY® incorporates into cell membranes and responds to lipid peroxidation by going through a spectral emission shift from red to green [54]. The nucleic acid stain, Propidium Iodide, only penetrates membranes of compromised cells and subsequently binding to nuclear DNA. Samples were loaded with BODIPY® (1 µM) for 30 min, centrifuged at 2300 g for 5 min, then exposed to the various treatments (see below), then stained with Propidium Iodide (10 µM).

Immediately following staining, cells were analyzed by flow cytometry that was performed using an Accuri C6 flow cytometer (Accuri Cytometers, Inc. Ann Arbor, MI USA) and data analyzed using the Accuri software (CFlow Plus, Ver. 1.0.202.1). The C6 is equipped with a blue and a red laser, two scatter detectors, and four fluorescence detectors (FL1 533/30 nm; FL2 585/40 nm; FL3>670 nm and FL4 675/25 nm) whose range displayed data across 6.2 logs. Because of this, no pre-gating was required as the C6 has a digital data collection system that allowed it to display and analyze all possible data post-collection without any alteration of the original data file. Forward scatter and side scatter were plotted, as well as florescence detected by plotting detection on FL-1 versus FL-2. Gating and fluorescence compensation values were set after data collection.

In order to limit the evaluation of DHE and BODIPY® fluorescence to viable spermatozoa, the subpopulation of SYTOX® Green or propidium iodide-positive cells (membrane compromised cells) were removed from the evaluation of 10,000 events (cells) per sample. Sample cell numbers were determined by measuring the volume needed to achieve 10,000 events with the C6.

Along with the other treatment groups (detailed below), two controls were included in all experiments to help identify live and dead cells for the flow cytometer; 1) unstained, untreated cells; and 2) cells destroyed by 3 cycles of freeze-thawing followed by staining with DHE and SYTOX® Green or BODIPY® and propidium iodide.

Since the motility of activated zebrafish sperm lasted less than 1 min, their motility measurements must be taken quickly. A single observer determined the sperm motility with a phase contrast microscope (200 x, Olympus BX41), measuring the mean percent progressive motility. Specifically, two µl of sperm at 10^7^ cells/ml were placed onto the surface of a slide, 18 µl of deionized water was added to activate the sperm, the drop was gently mixed on the slide, and the motility measured within 5 to 10 sec of mixing. The slide was moved to assess at least 3 full frames of sperm motility and estimated at <10, 25, 50, 75 or > 90% progressive motility.

### Experiment 1: Establishing of the Xanthine-Xanthine Oxidase (X-XO) System and DHE Method to Evaluate ROS Production with Zebrafish Spermatozoa

Before examining ROS production in zebrafish sperm cryopreservation, we established ROS production using a known ROS generating system. The X-XO system was used to generate the reactive oxygen species, superoxide and hydrogen peroxide. An aliquot (250 µl at 1×10^7^ cells/ml) of the sperm sample (N = 10) was treated with one of 3 doses of X-XO. The range of concentration or dose of X-XO used in McCarthy and Meyers [40] served as a guide for our preliminary experiments. The treatments contained: (1) sperm treated with DHE (control), (2) low X-XO (0.1 mM X and 0.01units/ml XO), (3) medium X-XO (0.2 mM X and 0.0175 units/ml XO) and (4) high X-XO (0.3 mM X and 0.025 units/ml XO). Treated samples were incubated for 30 min then a small sample was taken for motility analysis, as described above. The remaining samples were stained with DHE and SYTOX® Green and analyzed on the flow cytometer (see above) for cell viability and oxidative stress.

### Experiment 2: Mitigation of ROS by Candidate Anti-Oxidants

The addition of scavengers for reactive oxygen species, SOD, CAT and Vitamin E was examined to determine whether they could decrease ROS. After squeezing males, the concentration of spermatozoa was adjusted to 1×10^7^/mL in 250 µl aliquots and pre-treatment motility evaluated. The samples were then incubated at room temperature for 30 min, according to the following treatments: (1) sperm treated (control), (2) X-XO (0.2 mM X and 0.0175 U/ml XO), (3) SOD (200 U/ml), (4) X-XO (0.2 mM X and 0.0175 U/ml XO) + SOD (100 U/ml), (5) X-XO (0.2 mM X and 0.0175 U/ml XO) + SOD (200 U/ml), (6) Vitamin E (100 µM), (7) X-XO (0.2 mM X and 0.0175 U/ml XO) + Vitamin E (100 µM), (8) X-XO (0.2 mM X and 0.0175 U/ml XO) + Vitamin E (200 µM), (9) CAT (200 U/ml), (10) X-XO (0.2 mM X and 0.0175 U/ml XO) + CAT (100 U/ml), (11s) X-XO (0.2 mM X and 0.0175 U/ml XO) + CAT (200 U/ml). After the 30 min treatment- exposure, a sub-sample was taken from each treatment for motility assessments. The remaining sample was stained with DHE for 10 minutes, followed by SYTOX® Green for 5 minutes, then examined on the flow cytometer to measure the number of DHE (ROS)- and SYTOX® Green-positive cells (viability), as described above.

### Experiment 3: Evaluation of Cryopreservation Stress and the Subsequent Effect on ROS Production with Zebrafish Spermatozoa

Cells were subjected to cryopreservation stress to determine (1) if the stress resulted in the production of ROS and (2) if CAT could mitigate stress contributed by hydrogen peroxide, or increase viability or motility over control values. After squeezing males, sperm samples were diluted to either 1×10^7^ cells/ml or 2×10^7^ cells/ml, depending on their treatment. Samples treated with cryoprotectant were diluted 1∶1, so an initial cell concentration of 2×10^7^ cells/ml was used in these samples. The following sperm treatments were analyzed using DHE or BODIPY®): (1) sperm treated (control), (2) CAT (200 U/ml), (3) 8% methanol, (4) 8% methanol + CAT (200 U/ml), (5) cryopreserved sperm with 8% methanol, (6) cryopreserved sperm with 8% methanol + CAT (200 U/ml) added pre-freeze. Preliminary experiments determined that CAT added prefreeze instead of post-thaw, produced the best results. Cryopreservation was performed as described by Yang and coworkers [6]. Briefly, sperm were incubated for 10 minutes on ice in an 8% methanol solution in HBSS, loaded into 0.25 ml French straws, then frozen at a rate of 10°C/min from 4°C to −80°C, using a Planer Kryo 320 - 1.7 controlled rate freezer (Planer PLC, Middlesex, UK). Sperm samples were then quenched in liquid nitrogen for at least 10 minutes before thawing (20 to 40 sec at 30°C). Motility was assessed immediately following the 10 min cryoprotectant exposure (for fresh samples) or post-thaw, for frozen samples. Samples were stained with DHE and SYTOX® Green (as described in Experiment 2) or BODIPY® and propidium iodide (as described above) and assessed on the flow cytometer for viability and ROS. Each sample consisted of fresh, pooled sperm from at least 3 males (N = 9 to 13 samples/treatment, except for one CAT treatment with an N = 5). Additionally, preliminary cryopreservation experiments were also performed to determine (1) if the stress resulted in the production of ROS and (2) if SOD (200 U/ml) could mitigate stress contributed by superoxide (as measured by DHE fluorescence), or increase viability or motility over control values (N = 5 samples/treatment).

### Experiment 4: Evaluating Other Cryoprotectants on Motility and their Response to Antioxidants

To determine whether other cryoprotectants might be better candidates than methanol in terms of the stress they cause to the sperm, we compared 8% methanol, 8% methanol + CAT, 10% DMA, 10% DMA + CAT, 10% DMF and 10% DMF + CAT. These cryoprotectants were chosen because they have worked with zebrafish sperm to produce viable sperm post-thaw [3]–[6]. Each sample consisted of fresh, pooled sperm from at least 3 males. The following sperm-treatment groups were examined: (1) untreated sperm, N = 16; (2) CAT, N = 16; (3) 8% methanol, N = 16; (4) 8% methanol + CAT, N = 16; (5) 10% DMF, N = 8; (6) 10% DMF + CAT, N = 8; (7) 10 % DMA; and, (8) 10 % DMA + CAT, N = 8. The samples were held for 10 min at 0°C, then it’s motility assessed on a phase microscope (200 x as described above). The concentration of the CAT used in these experiments was (200 U/ml).

### Data Analysis

All data analyses in this study were performed using Graphpad Prism 5.0 (San Diego, CA) and Microsoft Excel (version 2007). All percent data were either LOG or arcsine transformed, and difference in the means was analyzed with either a one-way analysis of variance and a Tukey’s Multiple Comparison test or with a Kruskal-Wallis test and a Dunn’s Multiple Comparison test. A value of *P*<0.05 was considered significant, and all data was expressed as mean ± SE unless otherwise stated.

## Results

### Experiment 1: Establishing of the Xanthine-Xanthine Oxidase System and Methods to Evaluate ROS Production with Zebrafish Spermatozoa

**Figure 1:**
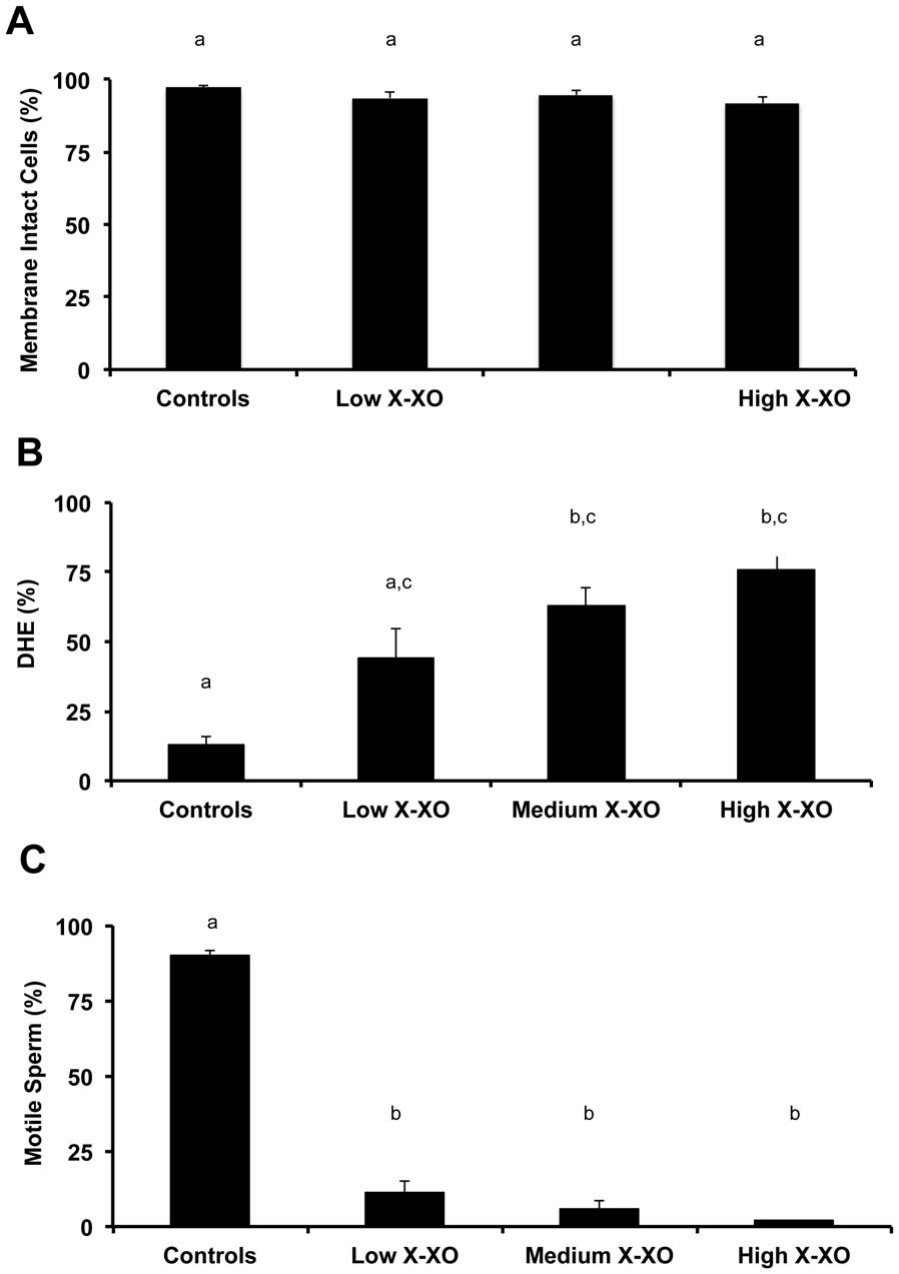
Evaluation of the xanthine-xanthine oxidase (X-XO) system and methods to identify DHE production (general oxidative stress) during ROS in zebrafish spermatozoa. In a concentration dependent manner, induced oxidative stress (X-XO): (**A**) did not alter the membrane integrity or viability (as assayed by propidium iodide) from the control values (P>0.05; Kruskal-Wallis test and Dunn’s Multiple Comparison test); (**B**) increased stress as assayed by DHE-positive cells (P<0.05; Kruskal-Wallis test and Dunn’s Multiple Comparison test); and, (**C**) reduced sperm motility as assayed by visual inspection with phase microscope (P<0.05; Kruskal-Wallis test and Dunn’s Multiple Comparison test). Bars with the same letter are not significantly different (P>0.05), but bars with different letters are (P<0.05). Low X-XO exposure  =  0.1 mMX + 0.01 units/ml XO; Medium X-XO exposure  =  0.1 mMX + 0.01 units/ml XO; High X-XO exposure  =  0.3 mM X + 0.025 units/ml XO. In all treatments, N = 10 samples and each sample consisted of pooled sperm from 3 to 5 males.

The induced oxidative stress (X-XO) system was functional for zebrafish sperm. In these studies, X-XO- generated oxidative stress was evaluated using DHE as an indicator of the superoxide anion. An increase in percentage of cells expressing high levels of DHE indicated increasing superoxide anion and, hence, oxidative stress in the system. The X-XO system was capable of producing a stress reaction in zebrafish sperm in a concentration dependent manner, which correlated with decreased motility, but had no affect on cell viability (Fig. 1). For the remainder of the tests, a medium X-XO concentration (0.2 mM X and 0.0175 U/ml XO) was used.

### Experiment 2: Mitigation of ROS by Candidate Anti-Oxidants

**Figure 2:**
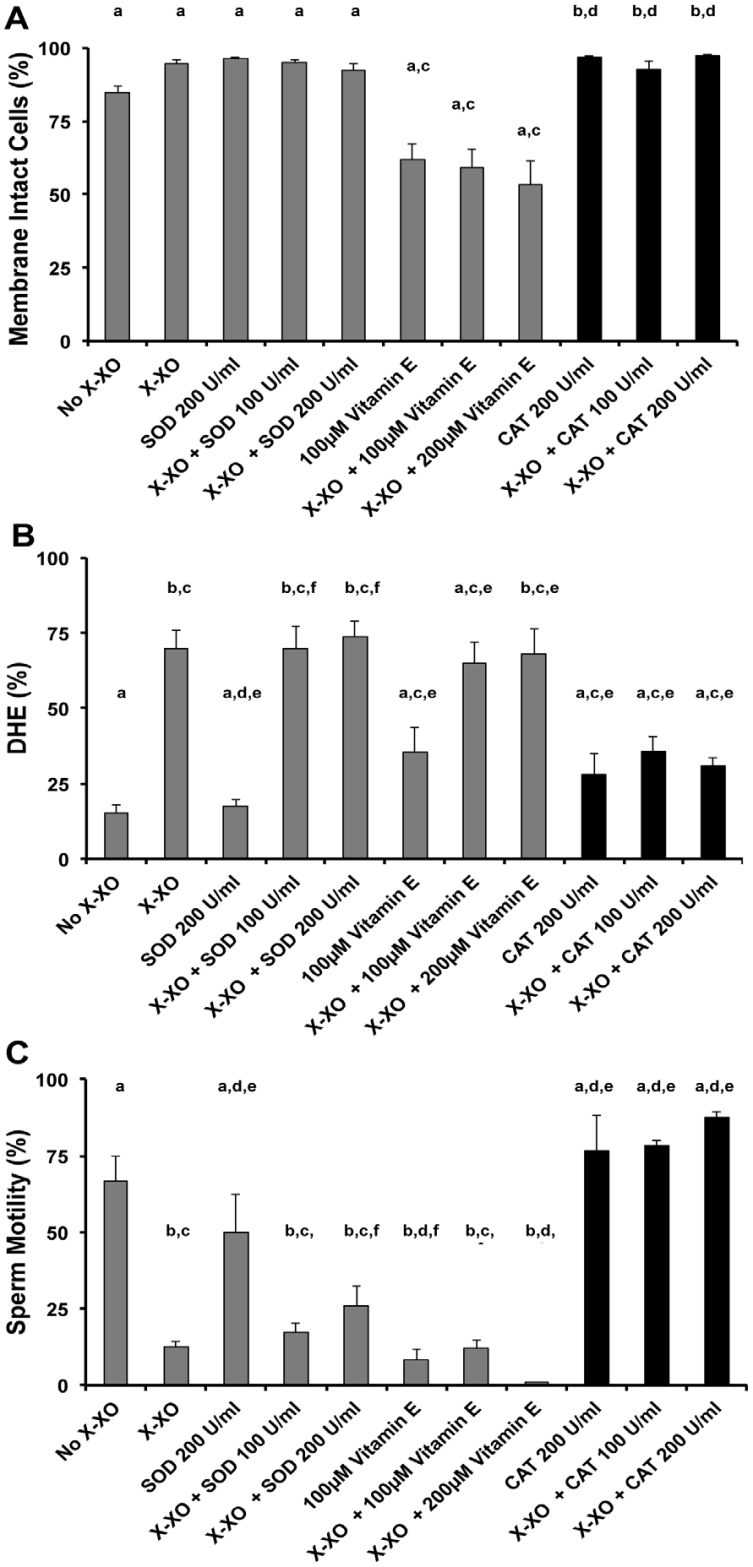
Evaluation of the xanthine-xanthine oxidase (X-XO) system and the impact of antioxidants on sperm viability, ROS and motility. In all the various treatments, the addition of CAT (black bars) performed the best. (**A**) Viability was not altered by most of the treatments as their mean values were not different than the control values (P>0.05), except CAT 200 U/ml, X-XO + CAT 100 U/ml X-XO + CAT 200 U/ml which were slightly higher than controls (P<0.05, Kruskal-Wallis and Dunn’s Multiple Comparison test). (**B**) Only the combination of X-XO + CAT mitigated the oxidative stress as their values were not different than the control values (P>0.05), whereas the combinations of X-XO + SOD and X-XO + vitamin E did not (P<0.05, Kruskal-Wallis and Dunn’s Multiple Comparison test). (**C**) Increased stress was correlated with decreased sperm motility that was mitigated by X-XO + CAT (P>0.05), but not by X-XO + SOD or X-XO + vitamin E (P<0.05; ANOVA and Tukey’s Multiple Comparison test, F = 39.5). Bars with the same letter are not significantly different (P>0.05), but bars with different letters are different (P<0.05). The X- axis categories and sample sizes for all tests were: No X-XO  =  control (N = 4); X-XO (in all treatments)  = 0.2 mM X and 0.0175 U/ml XO (N = 11); SOD 200 U/ml (N = 4); X-XO + SOD 100 U/ml (N = 12); X-XO + SOD 200 U/ml (N = 12); 100 µM vitamin E (N = 9); X-X0 + 100 µM vitamin E (N = 9); X-X0 + 200 µM vitamin E (N = 9); CAT 200 U/ml (N = 4); X-XO + CAT 100 U/ml (N = 12); X-XO + CAT 200 U/ml (N = 12). In all trials, a medium X-XO exposure was used when X-XO was added. In all treatments, each sample consisted of pooled sperm from at least 3 males.

Overall, the addition of the X-XO and combinations of X-XO + SOD or X-XO + and vitamin E did not impact the cell viability (P>0.05, Kruskal-Wallis and Dunn’s Multiple Comparison test), but the CAT and X-XO + CAT treatments improved viability over control values (P<0.05, Kruskal-Wallis and Dunn’s Multiple Comparison test, Fig. 2A). The X-XO system increased DHE-positive cells from about 15% (control) to about 70% (X-XO) (P<0.05, Kruskal-Wallis and Dunn’s Multiple Comparison test), but only the addition of CAT mitigated this stress to control levels (P>0.05, Kruskal-Wallis and Dunn’s Multiple Comparison test, Fig. 2B). Additionally, only CAT maintained sperm motility at or slightly above control levels (P>0.05, Kruskal-Wallis and Dunn’s Multiple Comparison test, Fig. 2C), whereas X-XO alone or in combination with SOD or vitamin E produced over a 50% reduction in motility (Kruskal-Wallis, P<0.05, Fig. 2C). Due to the fact that CAT produced superior results with the zebrafish sperm X-XO system, it was used in the subsequent experiments.

### Experiment 3: Cryoprotectant Stress and Mitigation by CAT

**Figure 3:**
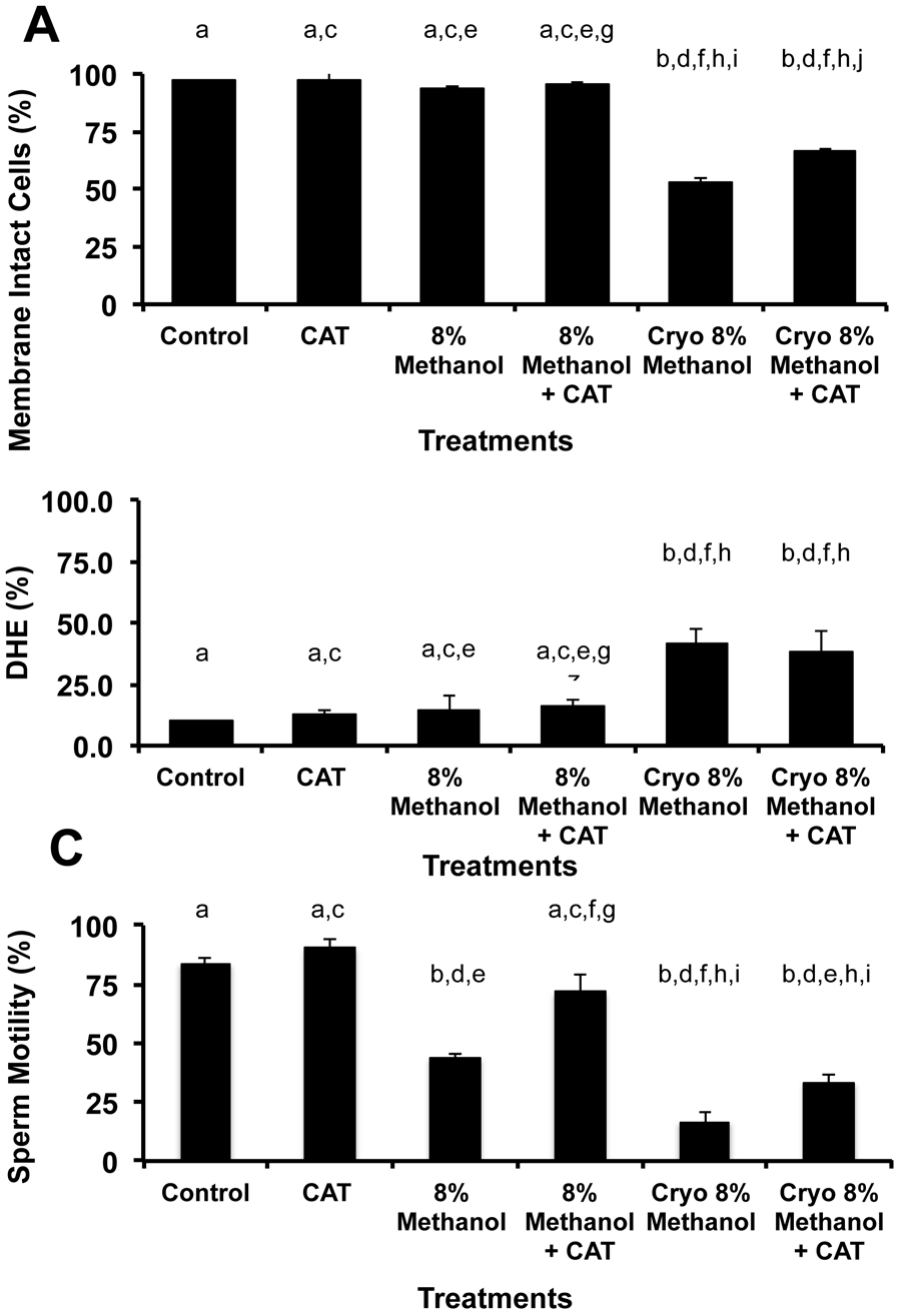
Evaluation of the intracellular ROS in zebrafish spermatozoa during cryopreservation and its effect on viability and motility. (**A**) Overall, cryopreservation decreased the viability of zebrafish sperm by approximately 50%, but the addition of CAT to the methanol improved its post-thaw viability 16% (P<0.05, ANOVA and Tukey’s Multiple Comparison test, F = 60.5). (**B**) Cryopreservation increased ROS over 2.7-fold (P<0.05, ANOVA, and Tukey’s Multiple Comparison test F = 6.2), but the addition of CAT did not mitigate it (P>0.5, ANOVA). (**C**) Motility demonstrated the greatest sensitivity in our treatments. Prior to cryopreservation, the 8% methanol treatment reduced motility by ∼50%, and the addition of CAT to the 8% methanol returned the motility to control levels. In parallel, the cryopreserved sperm (8% methanol) decreased the pot-thaw motility of zebrafish sperm from 83% (control) to 17%, however the addition of CAT doubled this post-thaw motility (P<0.05, ANOVA and Tukey’s Multiple Comparison test, F = 22.6). Bars with the same letter are not significantly different (P>0.05), but bars with different letters are (P<0.05). X- axis categories and sample sizes were: Control =  live sperm stained with DHE, N = 13; CAT (200 U/ml, concentration used in all CAT treatments), N = 9; 8% Methanol, N = 12; 8% Methanol + CAT 200, N = 12; Cryo 8% Methanol  =  sperm cryopreserved according to [6] with 8% methanol, N = 13; Cryo 8% Methanol + CAT, N = 13.

**Figure 4:**
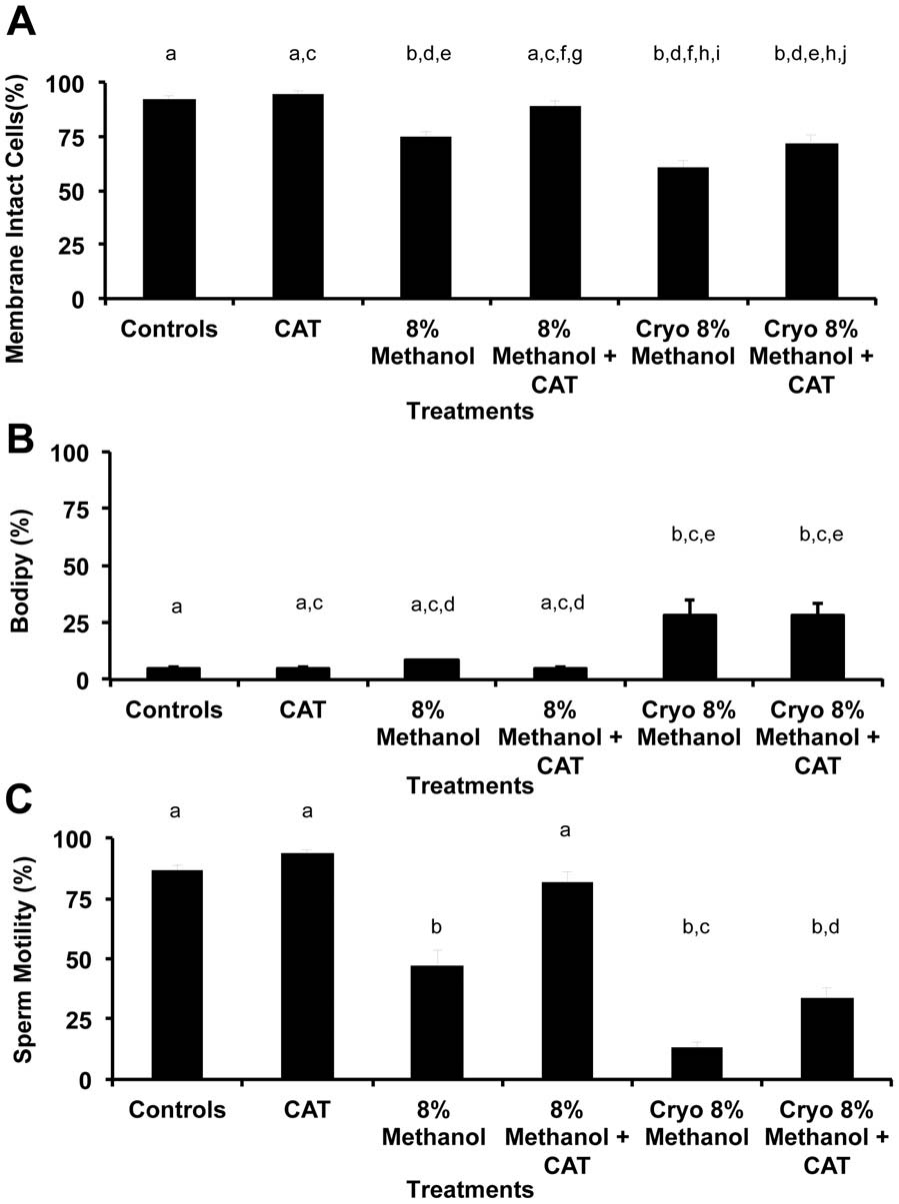
Evaluation of the lipid membrane ROS in zebrafish spermatozoa during cryopreservation and its effect on viability and motility demonstrated the same pattern as in Fig. 3. (**A**) Methanol (8%) decreased the viability of zebrafish sperm in fresh and cryopreserved treatments, and the addition of CAT to the methanol improved its viability in pre- and post cryopreservation treatments (P<0.05, ANOVA, F = 19.0). (**B**) Cryopreservation increased ROS 3.0-fold (P<0.05), but CAT did not mitigate it (P>0.5, ANOVA and Tukey’s Multiple Comparison test, F = 13.4). (**C**) The addition of 8% methanol decreased zebrafish sperm motility in fresh and cryopreserved treatments (P<0.05), and the addition of CAT to the methanol improved its viability in pre- and post cryopreservation treatments (P<0.05, ANOVA and Tukey’s Multiple Comparison test, F = 19.0). Bars with the same letter are not significantly different (P>0.05), but bars with different letters are (P<0.05). X- axis categories and sample sizes were: Control =  live sperm stained with DHE, N = 12; CAT (200 U/ml, concentration used in all CAT treatments), N = 5; 8% Methanol, N = 12; 8% Methanol + CAT 200, N = 12; Cryo 8% Methanol  =  sperm cryopreserved according to [6] with 8% methanol, N = 12; Cryo 8% Methanol + CAT, N = 12.

We examine both intracellular ROS and lipid peroxidation in zebrafish spermatozoa during cryopreservation and its correlated effects on viability and motility (Figs. 3 and 4). Several sperm treatments were examined including: (1) Control =  no treatment; (2) CAT (200 U/ml, concentration used in all CAT treatments); (3) 8% Methanol; (4) 8% Methanol + CAT; (5) cryopreservation with 8% Methanol; (6) cryopreservation with 8% Methanol + CAT.

Both intracellular ROS (Fig. 3) and lipid peroxidation (Fig. 4) showed similar responses to the treatments, except for a slight difference in the viability measurements. Specifically, ROS was increased 2.7(DHE)- to 3.0(BODIPY®)-fold after cryopreservation, (P<0.05), but this ROS was not mitigated by CAT (P>0.05, ANOVA). The addition of 8% methanol to fresh zebrafish sperm reduced its motility of (P<0.05, ANOVA), but adding 8% methanol + CAT maintained its motility at control levels (P>0.05, ANOVA) improving the post-thaw motility 2.5 (DHE)- to 2.9 (BODIPY®)-fold. Using DHE and SYTOX® Green to measure viability demonstrated a ∼50% loss in viability after cryopreservation with 8% methanol alone, but viability was improved 13% with the addition of CAT to the cryopreservation process (P<0.05, ANOVA, Fig. 3). Using BODIPY® and propidium iodide to measure viability both the fresh and cryopreserved sperm experienced a loss of viability that was improved with the addition of CAT to the process (P<0.05, ANOVA, Fig. 4).

Our preliminary results with SOD and cryopreservation did not vary from those reported here for CAT, except there was no improvement of post-thaw sperm motility with SOD (5%±2.2%) or without SOD (6%±2.2%).

### Experiment 4: Evaluating Other Cryoprotectants on Motility and their Response to Antioxidants

**Figure 5:**
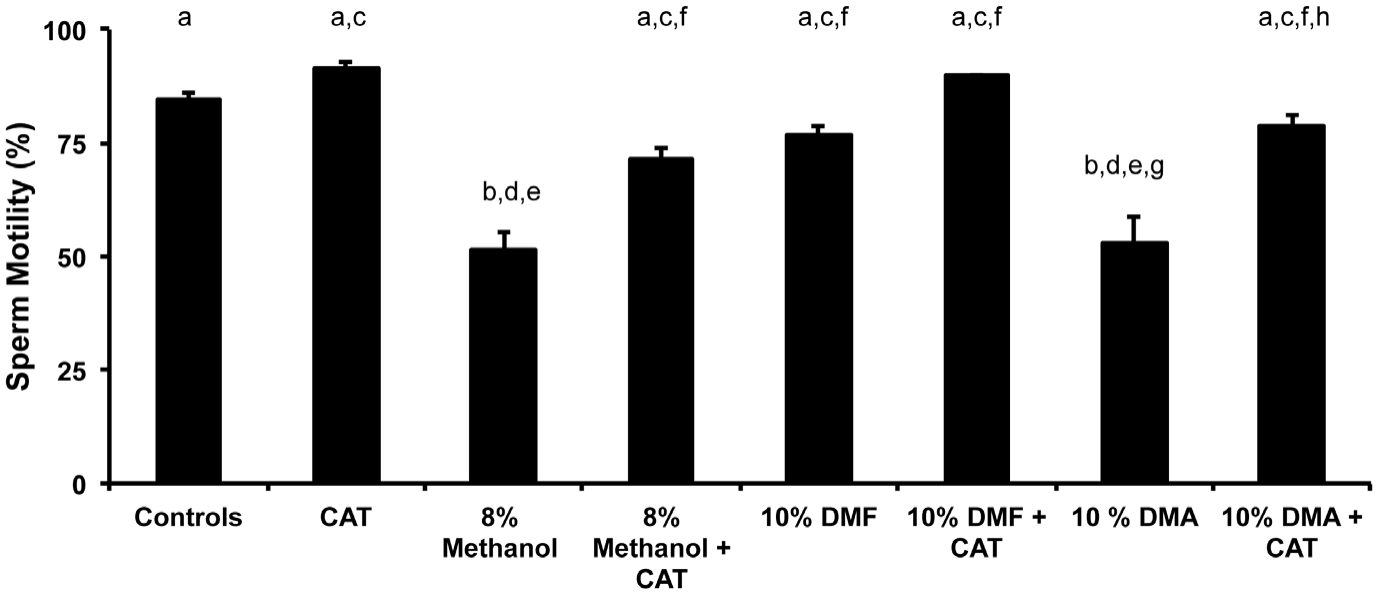
The effect of cryoprotectants and antioxidants on the motility of fresh zebrafish sperm. Each sample consisted of fresh, squeezed sperm pooled from 3 males that was treated with cryoprotectants alone or with cryoprotectants and CAT to examine the effect on the motility of the sample. X-axis categories and sample sizes were: Control, no treatment, N = 16; CAT (200 U/ml for all treatments), N = 16; 8% methanol, N = 16; 8% methanol + CAT, N = 16, 10% dimethylformamide (DMF), N = 8; 10% DMF + CAT, N = 8; 10% dimethylacetamide (DMA), N = 8; and, 10% DMA + CAT, N = 8. Of all the treatments, the 8% methanol and 10% DMA reduced the motility over the control value (P<0.5, ANOVA and Tukey’s Multiple Comparison Test, F = 15.4), whereas the other cryoprotectants (with or without CAT) did not (P>0.05). When CAT was added to the cryoprotectants, all the mean values increased, but the 10% DMF + CAT produced the highest mean motility at 90%. Bars with the same letter are not significantly different (P>0.05), but bars with different letters are (P<0.05). Thus, the addition of catalase had a beneficial effect on the motility of zebrafish sperm.

In zebrafish, pre-freeze and post-thaw motility is correlated fertilization success [2]. For successful cryopreservation, the process must begin with highest quality material possible, especially in regards to motility. Ideally, the addition of the cryoprotectant should not negatively impact the motility and viability of the sperm. However, in experiment 3, we observed that the routinely-used cryoprotectant (8% methanol) decreased motility and viability of the fresh sperm prior to the cryopreservation process. Some of this loss in motility was improved with CAT, but even with the help of CAT the motility values did not approach control values. To determine whether other cryoprotectants might impact the motility less, we examined 10% DMA and 10% DMF with and without CAT in addition to 8% methanol with and without CAT. In the fresh sperm treatments, both 10% DMF and 10% DMF + CAT maintained motilities at the control level (Fig. 5, P>0.05, ANOVA), whereas all the other treatment pairs did not (P<0.05, ANOVA, F = 15.4).

## Discussion

Preservation of genetic resources from a variety of animal disease models is paramount for maintenance of genetic diversity and the study of disease. The sheer numbers of genetically unique zebrafish lines makes systematic preservation of these models essential to preserve this valuable resource. There are simply too many such genetic lines to warrant maintenance of live animals for the entire resource. A long-term goal for this resource has been to preserve gametes, embryos, and stem cells since numerous methods will likely be necessary to ensure preservation of each of these resources. Cryopreservation of zebrafish sperm has been one method that has been used for several decades although research groups working with this resource have reported varying success rates.

Among vertebrates, successful cryopreservation of gametes has been shown to be highly variable and dependent on the species and conditions of cooling, media, and storage. Cryopreservation of sperm induces severe stresses in sperm cells owing largely to biophysical stressors such as intracellular and extracellular ice formation, osmotic stress, and oxidative stress. In our study, the positive response of zebrafish sperm to CAT, a mediator of hydrogen peroxide radicals, suggests that hydrogen peroxide could be the primary source of oxidative cell damage during cryopreservation. Although both the X-XO ROS generating system and the process of cryopreservation have been demonstrated to produce both superoxide anion and hydrogen peroxide in sperm cell systems in mammals, we found that a significant degree of mitigation of ROS-induced damage could result from the addition of the ROS scavenging enzyme, CAT, in comparison to the specific blockage of superoxide anion by SOD.

Mammalian sperm have been shown to be particularly susceptible to lipid peroxidation because they contain high concentrations of unsaturated fatty acids and because of the sperm cell’s inherent limited self-repair mechanism. Relatively little is known regarding the fatty acid composition of zebrafish sperm however, it seems clear from the present study that these cells are also highly susceptible to the effects from lipid peroxidation and oxygen free radicals. In previous studies, we have demonstrated that both equine and rhesus monkey sperm are susceptible to peroxidative damage resulting from cryopreservation [19], [55], [56] and that both superoxide anion and hydrogen peroxide are major sources of this damage. However, in both of these species it is apparent that hydrogen peroxide is the more significant of the two regarding ROS-induced cell damage. Especially since SOD had no effect on the post-thaw motility of the cryopreserved zebrafish sperm, suggesting that superoxide was not produced by cryopreservation. In porcine sperm, it has been demonstrated that superoxide anion plays a less significant role as a source of oxidation than does hydrogen peroxide [57].

An increase in ROS has been linked to abnormal or damaged spermatozoa in other species [10], [12], [14], [18]. It has also been reported in fish that ROS may induce DNA damage to sperm. For example, duroquinone-induced ROS caused DNA damage in carp sperm, which impaired reproductive success [15]. The X-XO system is one of several ROS generating systems frequently used to investigate the cytotoxic effects of the superoxide anion and hydrogen peroxide. Generation of ROS *in vitro* by the X-XO system results in reduced sperm motility [16]–[19], viability [16], [20], ionophore-induced acrosome reaction [18], and sperm-oocyte fusion [18], [22]. Hydrogen peroxide, in contrast to superoxide anion, is more stable and can readily cross the plasma membrane [58]. Consequently, hydrogen peroxide appears to be the primary ROS responsible for oxidative damage to spermatozoa *in vitro* in some systems [16], [19], [21], [22], [24]. Membrane lipid peroxidation also is believed to be an important mechanism of action [10], [18], [59]. Spermatozoa are particularly susceptible to this condition, in part, because they contain high fatty acid concentrations [27] and, as terminally differentiated cells, have limited repair mechanisms [28]. Lipid peroxidation causes loss of plasma membrane fluidity and integrity required for fusion events associated with fertilization [30], [59].

One of the goals of this work was to determine whether an *in vitro* ROS system could be established in zebrafish to examine cryopreservation-related stresses. In this way, a rational approach to minimizing the damage that sperm incur during cryopreservation can be determined. The antioxidant, CAT, performed best in our studies. It maintained viability and motility, and mitigated stress in the X-XO system, returning it to control levels. In contrast, SOD and vitamin E did not improve any measures of sperm viability/membrane integrity and motility. These studies may not be an accurate assessment of vitamin E, however, because it was dissolved in methanol, which may have negatively impacted its physiological action. Elevated BODIPY®-labeling of zebrafish sperm was associated with cryopreservation and X-XO induced ROS production in this study, suggesting that hydrogen peroxide production may be the dominant ROS in the zebrafish model.

Methanol is one of the main cryoprotectants for zebrafish sperm used in laboratories around the world [51]. Unfortunately, 8% Methanol performed very poorly in our experiments reducing sperm motility, and viability prior to the cryopreservation process (as measured by BODIPY® and propidium iodide). Most importantly, motility correlates with fertilization success in zebrafish [2]. Methanol, DMA and DMF are a class of cryoprotectants that do not cause osmotic swelling of zebrafish sperm during the solute equilibration process [60] which makes them excellent candidates for understanding and improving the cryopreservation process. However, 10% DMF maintained control levels of motility and viability, whereas 8% methanol and 10% DMA did not. These experiments did not address whether 10% DMF or 10% DMF + CAT would out perform 8% methanol or 8% methanol + CAT in improving fertilization success. This is because additional studies need to accomplished to examine all three cryoprotectants and CAT in parallel using the exact same procedures (i.e., sperm concentrations, buffers, *in vitro* fertilization assays etc.) to understand their strengths and weaknesses. The cryopreservation process did increase intracellular and lipid ROS, and CAT mitigated this by improving post-thaw motility and viability. However, we also observed that the addition of CAT did not decrease the ROS production after cryopreservation. This is puzzling considering CAT reduced ROS stress in the X-XO system. The reasons for these mixed observations remain unclear. Additional studies will determine the nature of lipid composition of fish sperm membranes and their susceptibility to membrane damage.

In summary, these experiments indicated that the X-XO system could be used for determination of zebrafish sperm oxidative damage and selection of appropriate antioxidants to help mitigate ROS during zebrafish sperm cryopreservation. Further studies will be necessary to evaluate other potential ROS and Reactive Nitrogen Species, including nitric oxide and others, and their possible roles in oxidation of membranes and DNA of zebrafish sperm. In addition, our study demonstrated that sperm from this aquatic species behaves similar to the more extensively studied mammalian sperm. Although we used an accepted cryopreservation protocol [6] for the cryopreservation experiments with the 8% methanol, this and many of the other related protocols will need improvement in the future. Certainly, the addition of CAT to present procedures may be able to more than double the post-thaw motility, assuring increased and less variable fertilization success for zebrafish laboratories and stock centers around the world.
